# Contemporary treatment of mitral valve disease with transcatheter mitral valve implantation

**DOI:** 10.1007/s00392-022-02095-y

**Published:** 2022-09-15

**Authors:** Hendrik Wienemann, Victor Mauri, Laurin Ochs, Maria Isabel Körber, Kaveh Eghbalzadeh, Christos Iliadis, Marcel Halbach, Thorsten Wahlers, Stephan Baldus, Matti Adam, Elmar Kuhn

**Affiliations:** 1 Faculty of Medicine and University Hospital Cologne, Clinic III for Internal Medicine, University of Cologne, Kerpener Str. 61, 50937 Cologne, Germany; 2 Faculty of Medicine and University Hospital Cologne, Department of Cardiothoracic Surgery, University of Cologne, Kerpener Str. 61, 50937 Cologne, Germany

**Keywords:** Mitral valve disease, Transcatheter mitral valve implantation, Tendyne, Valve-in-valve, Heart valve prosthesis

## Abstract

**Background:**

Transcatheter mitral valve implantation (TMVI) with self-expanding (SAV) or balloon-expandable (BAV) valves are rising as promising treatment options for high-risk patients with symptomatic mitral valve (MVD) disease unsuitable for alternative treatment options.

**Aims:**

The aim of this study was to examine the clinical, procedural and outcome parameters of patients undergoing SAV or BAV for MVD.

**Methods:**

In this observational and single-center case series, fifteen consecutive patients treated with the Tendyne Mitral Valve System (SAV) and thirty-one patients treated with SAPIEN prosthesis (BAV) were included.

**Results:**

The patients (aged 78 years [interquartile range (IQR): 65.5 to 83.1 years], 41% women, EuroSCORE II 10.3% [IQR: 5.5 to 17.0%] were similar regarding baseline characteristics, despite a higher rate of prior heart valve surgery and prevalence of MV stenosis in the SAV-group. At discharge, the SAV-group had a mean transvalvular gradient of 4.2 mmHg, whereas the BAV-group had a mean transvalvular gradient of 6.2 mmHg. None or trace paravalvular leakage (PVL) was assessed in 85% in SAV-group and 80% in the BAV-group. 320 day all-cause and cardiac mortality rates were comparable in both groups (SAV: 26.7% vs BAV: 20%, *p* = 0.60). Four deaths occurred early in the SAV-group until 32 days of follow-up.

**Conclusions:**

In high-risk patients with MVD, TMVI presents a promising treatment option with encouraging mid-term outcomes and good valve durability. TMVI either with BAV or SAV may be developed to an established treatment option.

**Graphical abstract:**

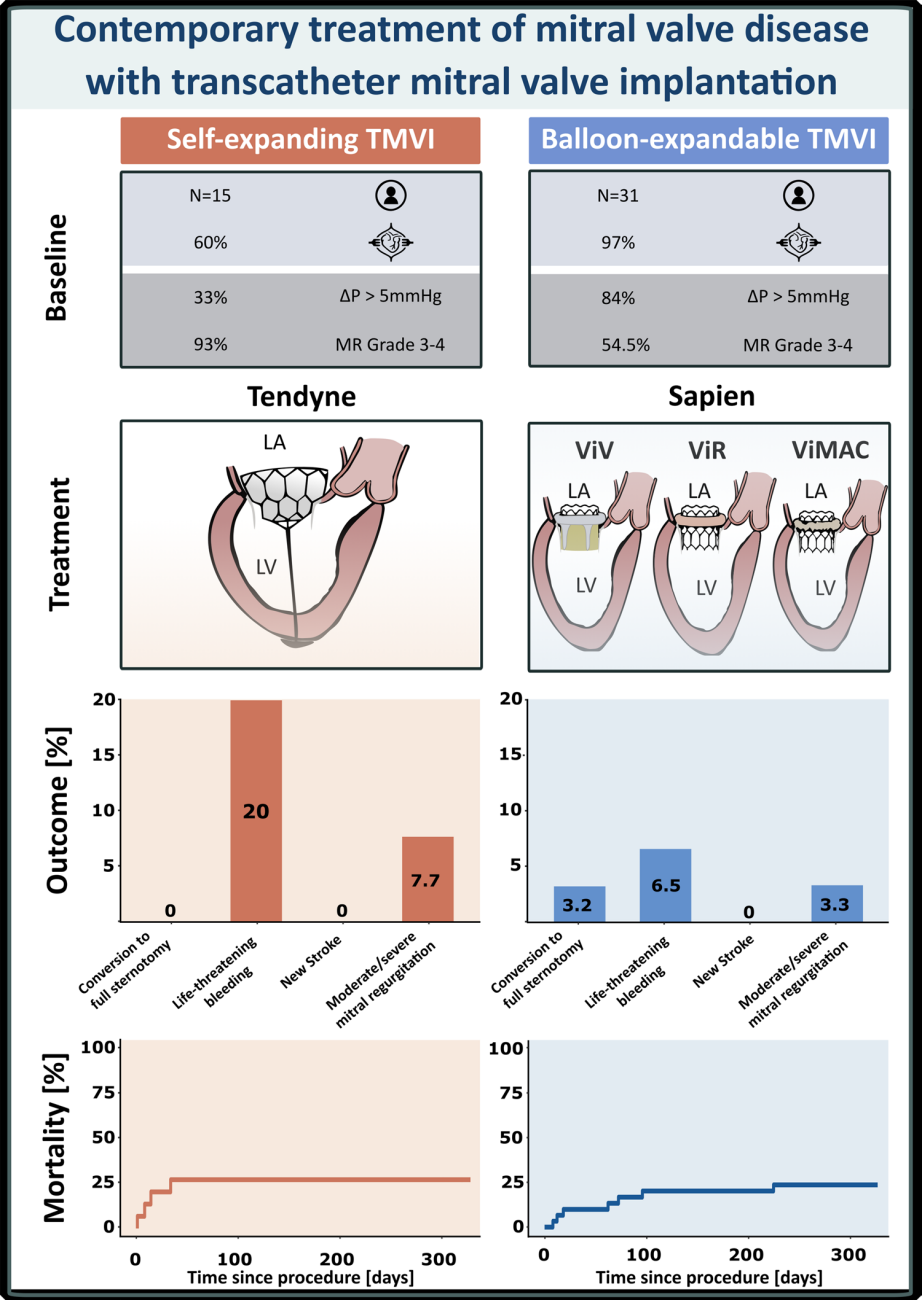

## Introduction

Mitral valve disease (MVD) is linked to a high morbidity and mortality [1]. Hitherto surgical valve repair or replacement represents the main treatment option [2, 3]. However, in a significant proportion of patients surgery is accompanied by risks due to extensive comorbidities including prior cardiac surgery. For these high-risk patients, catheter-based treatment options have been introduced into clinical practice. For patients unsuitable for mitral transcatheter edge-to-edge repair (M-TEER) or annuloplasty, transcatheter mitral valve implantation (TMVI) and transapical mitral valve implantation are potential therapeutic options. However, the value of these new treatment options needs to be assessed.

Currently, the balloon-expandable SAPIEN transcatheter heart valve (BAV, Edwards Lifesciences, Irvine, CA, USA) can be implanted in patients with bioprosthetic valve failure as valve-in-valve (VIV), in patients with ring failure as valve-in-ring (ViR) or can be implanted in mitral annular calcification (MAC) as valve-in-MAC (ViMAC) [4–6].

In addition, a growing number of dedicated transcatheter prostheses is available for transcatheter mitral valve implantation (TMVI) offering an alternative for patients with MVD and suitable anatomy. The Tendyne system (Abbott Cardiovascular, Plymouth, MN, USA) offers one promising self-expanding valve (SAV) technology. Recently, the 1- and 2-year follow-up data showed promising results with a sustained reduction in mitral regurgitation (MR) [7, 8]. But currently, direct real-world experience of these different treatment options in high-risk patients with MVD is sparse.

We sought to evaluate TMVI with SAV and BAV regarding clinical, hemodynamic, and echocardiographic outcomes in patients with mitral valve disease.

## Methods

### Study Population

This study included all consecutive patients undergoing either BAV (Edwards Lifesciences SAPIEN prosthesis) or SAV (Tendyne Mitral Valve System) between 12/2014 and 10/2021 at Cologne University Heart Center. Patients were symptomatic despite receiving efficient guideline-directed medical treatment and judged as high-risk for conventional mitral valve (MV) surgery by the multidisciplinary heart team but were eligible for TMVI. Patients undergoing concurrent intervention in the same procedure were excluded. Baseline demographic and clinical data were obtained from electronic medical records and recorded in a dedicated database.

### Preoperative planning

All patients had transthoracic and transesophageal echocardiography to grade MR regurgitation (no/trace, mild, moderate, or severe) and mitral stenosis (MS) according to guideline recommendation [3, 9, 10]. The following parameters were measured: left ventricular ejection fraction, left ventricular end diastolic diameter, mean transvalvular pressure gradient, and pulmonary artery systolic pressure (PASP). Right ventricular function was measured by tricuspid annular plane systolic excursion. The criteria of the European Association of Echocardiography and the American Society of Echocardiography were used to describe the mechanism of bioprosthetic valve or ring failure [10]. Mixed failure was classified as having at least mild MR and MS. Coronary arteries were assessed either by invasive or computed tomography (CT) coronary angiography. Contrast-enhanced multidetector CT imaging was used for procedural planning using 3mensio software (3mensio Structural Heart, 3mensio Medical Imaging, Maastricht, The Netherlands) with a focus on annulus geometry, access assessment, left ventricular outflow tract (LVOT) size, mitral annular calcification (MAC) and left ventricular size. MAC was classified according to the definition by Guerrero et al. as none, mild, moderate or severe [11].

### TMVI procedure with BAV

TMVI was performed through either a transseptal or transapical access. All patients in the BAV-group for ViV, ViR and ViMAC treatment underwent TMVI with the balloon-expandable SAPIEN XT, SAPIEN 3 or SAPIEN 3 Ultra (Edwards Lifesciences, Irvine, CA, USA). Balloon valvuloplasty before and after TMVI was performed at the discretion of the treating physicians. The patients were given antiplatelet treatment or anticoagulation in case of long-term anticoagulation indication before TMVI.

### The Tendyne Mitral valve System and procedure

The Tendyne Mitral Valve System (Abbott Cardiovascular, Plymouth, MN, USA) is the only TMVI system with CE-mark. It consists of double frame device with a tether anchored to an apical pad [12]. An outer sealing stent incorporates a circular inner stent that contains a trileaflet self-expanding trileaflet porcine valve prosthesis sutured to a double nitinol frame. Different sizes and profiles are available to cover different anatomic conditions. The self-expanding Tendyne Mitral Valve Device is implanted under general anesthesia via a transapical approach inserting a 36-F sheath. The prosthesis is implanted in the native MV annulus with the tether connected to an epicardial pad over the apical puncture site without the need for rapid pacing. All patients were given oral anticoagulation with a vitamin K antagonist (INR 2.0–3.0) after the operation.

### Data acquisition and follow‑up

Baseline, procedural, discharge information and survival data were collected during routine clinical practice using internal data and reported according to the Mitral Valve Academic Research Consortium (MVARC) standards [13]. Minor additions were made similar to Guerrero et al. Technical success at exit from hybrid operating room was defined as successful delivery and retrieval of the transcatheter delivery system via transapical or transfemoral access, deployment of a single valve in the correct position in the mitral annulus, no need for surgery or additional reintervention, and patient discharged alive from hybrid operating room. Device success at 30 days was defined as absence of mortality or stroke; and no migration, fracture, thrombosis, hemolysis or endocarditis with original valve in proper position; and freedom from unplanned surgical or interventional procedures linked to the device or access procedure; and a mean MV gradient < 10 mm Hg and residual MR less than moderate [14–16]. All patients gave informed consent for the procedures. The study was conducted in accordance with the principles contained in the Declaration of Helsinki and Good Clinical Practice guidelines. Approval was obtained from the institutional ethics board (22–1057). Follow-up was conducted according to clinical indications.

### Statistical analysis

Continuous variables are presented as mean ± standard deviations or as median with interquartile range (IQR) from the 25 to 75th percentiles, if data were not normally distributed. Normal distribution was tested with QQ-plots and Shapiro–Wilk’s test. Categorical variables are shown as absolute values and percentages. Wilcoxon rank-sum test was applied for parametric group comparison. Patients were censored at death, at last follow-up or 320 days post implantation whichever occurred first. End points were estimated using Kaplan–Meier technique. The log-rank test was used to compare the groups. A two-tailed *p* value of < 0.05 was considered as statistically significant. Statistical analysis was conducted in SPSS Statistics (Version 27, IBM, Armonk, New York) and R environment (R version 4.1.3, R Foundation for Statistical Computing, Vienna, Austria).

## Results

### Baseline characteristics of patients

A total of forty-six patients were included. Median age of the entire cohort was 77.8 (IQR 65.5, 83.1) years and 59% (*n* = 27) were males. 90% of the patients presented with NYHA class III or IV. Comorbidities are displayed in (Table 1a). These conditions resulted in increased surgical risk of 10.3% (IQR 5.5, 17.0) according to the EuroSCORE II. Both treatment groups were similar regarding baseline comorbidities. Naturally, prior heart surgery was different with thirty patients (97%) in the BAV-group and in nine patients (60%) in the SAV-group. Additionally, more patients in the BAV-group (*n* = 13, 42%) had MV stenosis compared to SAV-patients (*n* = 0). In the BAV-group, one patient had severe MAC, nine patients (29%) presented with failed surgical rings, and twenty-one patients (68%) had deteriorated bioprosthetic valves. In the SAV-group, one patient had prior implantation of surgical ring, one patient underwent prior transcatheter edge-to-edge therapy and five patients had moderate or severe MAC (Table 1a). Severe mitral regurgitation was the treatment indication in ten (67%) out of fifteen patients in the SAV-group. Patients treated with BAV had higher mean pressure gradients than in the SAV-group (8.0 mmHg [IQR 6.0–10.3] vs 3.6 mmHg [IQR 3.0, 6.3]). Details regarding the echocardiographic parameters are provided in (Table 1b).

**Table 1:** (a) Patient characteristics, (b) Echocardiographic characteristics

	Overall, *N* = 46	BAV, *N* = 31	SAV, *N* = 15
(a)			
Age (years)	77.80 (65.52, 83.10)	73.90 (60.25, 81.35)	80.30 (72.75, 84.40)
Female Sex	19/(41%)	14/(45%)	5/(33%)
New York heart association functional class			
I	0/(0%)	0/(0%)	0/(0%)
II	5/(11%)	3/(9.7%)	2/(13%)
III	32/(70%)	21/(68%)	11/(73%)
IV	9/(20%)	7/(23%)	2/(13%)
EuroSCORE II	10.33 (5.51, 17.04)	11.95 (5.89, 17.30)	9.55 (5.25, 12.34)
Body mass index (kg/m^2^)	24.75 (22.42, 28.37)	24.80 (23.30, 28.50)	24.50 (22.20, 27.30)
Diabetes	14/(30%)	10/(32%)	4/(27%)
Coronary artery disease	26/(57%)	17/(55%)	9/(60%)
Glomerular filtration rate mL/kg/1.73m^2^	43.67 (23.76)	48.03 (25.64)	34.67 (16.66)
Dialysis	4/(8.7%)	3/(9.7%)	1/(6.7%)
Chronic obstructive pulmonary disease	8/(17%)	6/(19%)	2/(13%)
Peripheral vascular disease	7/(15%)	4/(13%)	3/(20%)
Rhythm			
Sinusrhythm	22/(48%)	16/(52%)	6/(40%)
Atrial fibrillation	12/(26%)	7/(23%)	5/(33%)
Pacemaker	12/(26%)	8/(26%)	4/(27%)
Prior left bundel branch block	6/(13%)	5/(16%)	1/(6.7%)
History of atrial fibrillation or flutter	25/(54%)	14/(45%)	11/(73%)
Prior stroke or transient ischaemic attack	3/(6.5%)	2/(6.5%)	1/(6.7%)
Prior heart surgery	39/(85%)	30/(97%)	9/(60%)

	Overall, *N* = 46	BAV, *N* = 31	SAV, *N* = 15
(b)			
Predominant valve pathology			
Mixed	19/(41%)	14/(45%)	5/(33%)
Regurgitation	14/(30%)	4/(13%)	10/(67%)
Stenosis	13/(28%)	13/(42%)	0/(0%)
Left ventricular ejection fraction			
> 50%	31/(67%)	20/(65%)	11/(73%)
41–50%	3/(6.5%)	2/(6.5%)	1/(6.7%)
31–40%	4/(8.7%)	3/(9.7%)	1/(6.7%)
< 31%	8/(17%)	6/(19%)	2/(13%)
Left ventricular end-diastolic diameter, cm	5.29 (0.76)	5.20 (0.81)	5.47 (0.65)
Mitral valve mean gradient, mmHg	6.70 (3.70, 9.93)	8.00 (6.00, 10.30)	3.60 (2.95, 6.30)
Mitral regurgitation			
Severe	29/(63%)	15/(48%)	14/(93%)
Moderate	2/(4.3%)	2/(6.5%)	0/(0%)
Mild	6/(13%)	5/(16%)	1/(6.7%)
None-trace	9/(20%)	9/(29%)	0/(0%)
Transvalvular gradient > 5 mmHg	31/(67%)	26/(84%)	5/(33%)
Transvalvular gradient > 10 mm Hg	12/(26%)	11/(35%)	1/(6.7%)
Severe tricuspid regurgitation	14/(30%)	8/(26%)	6/(40%)
Pulmonary artery systolic pressure, mmHg	55.00 (46.00, 59.00)	54.00 (47.25, 63.50)	55.00 (45.50, 58.00)
Systolic pulmonary artery pressure > 35 mmHg	40 / (87%)	25 / (81%)	15 / (100%)

Values are reported as mean ± SD for parametric variables, median (interquartile range) for nonparametric continuous variables, and *n* (%) for categorical variables, *BAV* balloon-expandable valve, *SAV* self-expanding valve

### Procedural characteristics

Transapical access was chosen in four patients in the BAV-group out of 31 (13%). All procedures in the BAV-group were performed utilizing SAPIEN prostheses. SAPIEN 3 or S3 Ultra were used in 27 (87%) patients, whereas four patients (13%) received a SAPIEN XT. Technical success was achieved in all procedures (100.0%). Procedural characteristics for each patient are displayed in (Tables 2, 4a, b). Total procedural time in the SAV-group was longer compared to the BAV-group (145 [IQR 127, 217] vs. 93 [IQR 68, 120] minutes). In contrast, fluoroscopic time was shorter in the SAV- compared to the BAV-group (8.5 [IQR 6, 16] vs. 22 [IQR 14, 28] minutes, respectively). After the procedure, all patients were transferred to intensive care units.

**Table 2:** Procedural Characteristics

	Overall, *N* = 46	BAV, *N* = 31^1^	SAV, *N* = 15
Access route			
Tansfemoral	27/(59%)	27/(87%)	0/(0%)
Transapical	19/(41%)	4/(13%)	15/(100%)
Procedure type			
TAVI in surgical MV	21/(45.7%)	21/(68%)	0/(0%)
TAVI/Tendyne in Ring	10/(21.7%)	9/(29%)	1/(6.7%)
TAVI/Tendyne in moderate/severe MAC	6/(13.0%)	1/(3%)	5/(33.3%)
TAVI/Tendyne in non severe MAC	9/(19.6%)	0/(0%)	9/(60%)
Anesthesia			
General anesthesia	44/(96%)	29/(94%)	15/(100%)
Conscious sedation	2/(4.3%)	2/(6.5%)	0/(0%)
Predilatation performed	18/(39%)	14/(45%)	4/(27%)
Postdilatation performed	4/(8.7%)	4/(13%)	0/(0%)
Total procedure time (min)	104.00 (77.00, 145.00)	93.00 (67.50, 120.00)	145.00 (127.25, 217.00)
Fluoroscopic time (min)	17.00 (10.00, 25.00)	22.00 (14.00, 28.00)	8.50 (6.00, 16.00)
Contrast Use (ml)	0.00 (0.00, 25.00)	14.00 (0.00, 29.50)	0.00

Values are reported as median (interquartile range) for nonparametric continuous variables, and n (%) for categorical variables, *BAV* balloon-expandable valve, *MAC* mitral annular calcification, *SAV* self-expanding valve

### Clinical outcomes and follow-up

#### Echocardiographic outcome

**Fig. 1:**
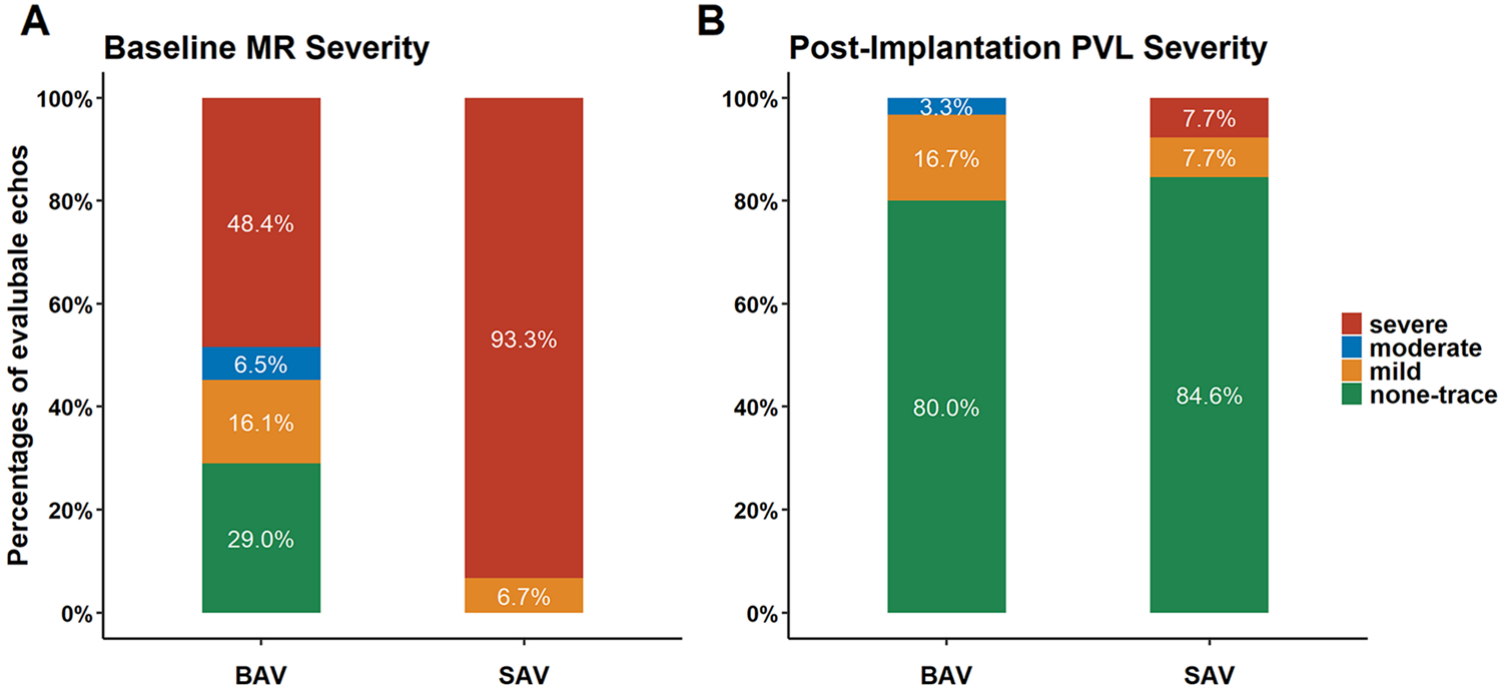
Mitral regurgitation (MR) severity was assessed at baseline (**A**) and paravalvular regurgitation (PVL) at discharge (**B**). At baseline, 55.0% of patients had MR moderate or severe in the group treated with a balloon-expandable valve. At discharge one patient of the fifteen (7.7%) treated patients with a self-expanding valve had severe PVL. *MR* mitral regurgitation, *BAV* balloon-expandable valve, *PVL* paravalvular regurgitation, *SAV* self-expanding valve

**Fig. 2:**
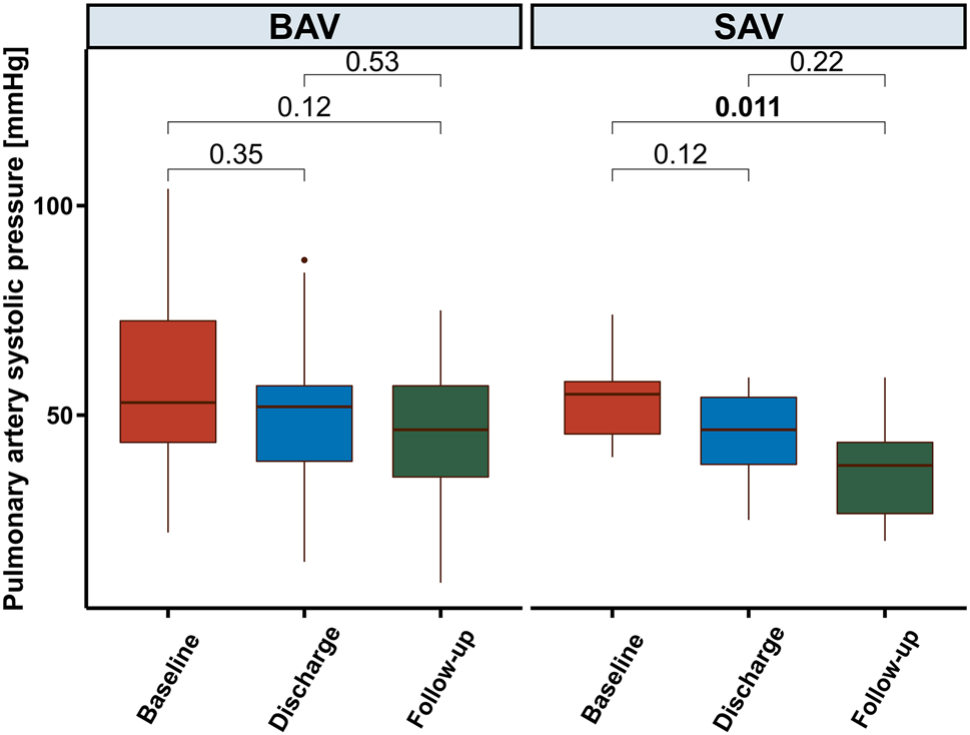
The mean systolic pulmonary artery pressure from baseline to follow-up after treatment with either a BAV (balloon-expandable valve) or SAV (self-expanding valve). Numerical decrease was observed in both groups, with statistical significance between baseline and follow-up for the SAV-group. *BAV* balloon-expandable valve, *SAV* self-expanding valve

80% of the patients had none or trace PVL in the BAV-group and 84.6% in the SAV-group (Fig. 1). One SAV-patient showed severe paravalvular regurgitation besides correct device positioning in the discharge assessment (Table 4a). Mean gradient after mitral (6.4 ± 2.0 vs 4.2 ± 2.6 mmHg) valve replacement was numerically higher in ViV/ViR group compared to the SAV-group (Table 3a). One patient showed a moderate paravalvular leak and one patient a mean transvalvular pressure gradient of 11 mmHg in the BAV-group (Table 4b). Lower PASP could be observed in the entire cohort, reaching statistical significance between baseline and follow-up in the SAV-group (Fig. 2).

**Table 3:** (a) Echocardiographic outcomes, (b) Procedural outcomes

	Overall, *N* = 46	BAV, *N* = 31	SAV, *N* = 15
(a)			
Mitral valve mean gradient, mmHg	5.78 (2.38)	6.40 (2.04)	4.23 (2.55)
Transvalvular gradient, mmHg	27/(64%)	23/(77%)	4/(33%)
Left ventricular end-diastolic diameter, cm	5.10 (0.76)	5.01 (0.75)	5.33 (0.79)
Pulmonary artery systolic pressure, mmHg	51.00 (38.00, 57.00)	52.00 (39.00, 57.00)	46.50 (38.25, 54.25)
Paravalvular regurgitation at discharge			
Severe	1/(2.3%)	0/(0%)	1/(7.7%)
Moderate	1/(2.3%)	1/(3.3%)	0/(0%)
Mild	6/(14%)	5/(17%)	1/(7.7%)
None-trace	35/(81%)	24/(80%)	11/(85%)
Paravalvular regurgitation at 30 days (*n* = 36)			
Severe	1/(2.8%)	0/(0%)	1/(10%)
Moderate	1/(2.8%)	1/(3.8%)	0/(0%)
Mild	5/(14%)	4/(15%)	1/(10%)
None-trace	29/(81%)	21/(81%)	8/(80%)

	Overall, *N* = 46	BAV, *N* = 31	SAV, *N* = 15	
(b)				
Conversion to open surgery	1/(2.2%)	1/(3.2%)	0/(0%)	
Bleeding				
None	35/(76%)	27/(87%)	8/(53%)	
Minor bleeding	1/(2.2%)	0/(0%)	1/(6.7%)	
Major bleeding	5/(11%)	2/(6.5%)	3/(20%)	
Life-threatening-bleeding	5/(11%)	2/(6.5%)	3/(20%)	
Vascular access complication				
None	36/(78%)	25/(81%)	11/(73%)	
Minor access complication	4/(8.7%)	3/(9.7%)	1/(6.7%)	
Major access complication	6/(13%)	3/(9.7%)	3/(20%)	
Rhythm at discharge				
Sinusrhythm	22/(48%)	16/(52%)	6/(40%)	
Atrial fibrillation	12/(26%)	7/(23%)	5/(33%)	
Pacemaker	12/(26%)	8/(26%)	4/(27%)	
Acute kidney injury requiring hemodialysis	4/(15%)	1/(8.3%)	3/(20%)	
New permanent pacemaker requirement	0/(0%)	0/(0%)	0/(0%)	
New stroke	1/(2.2%)	1/(3.2%)	0/(0%)	
In-hospital mortality	5/(11%)	2/(6.5%)	3/(20%)	
Intensive care unit stay, days	2.00 (1.00, 5.00)	2.00 (1.00, 4.00)	2.00 (1.50, 5.50)	
Total hospital stay, days	13.00 (8.00, 17.75)	10.00 (7.50, 15.50)	16.00 (11.00, 18.50)	

Values are reported as mean ± SD for parametric variables, median (interquartile range) for nonparametric continuous variables, and *n* (%) for categorical variables, *BAV* balloon-expandable valve, *MAC* mitral annular calcification, *SAV* self-expanding valve

**Table 4:** (a) Summary of individual Patient Data treated with Tendyne Device, (b) Summary of individual Patient Data treated with Sapien Device

Patient #	Sex	Age (years)	Mean MVG	MR-Pathology	MAC	Access	Prior bioprosthesis/ring	Implanted prosthesis	Technical success	Device success	Alive at 30 days
(a)											
1	M	79	6	Mixed	Severe	TA	–	LP-29L	Yes	Yes	Yes
2	F	89	3.6	Mixed	Mild	TA	–	LP-29L	Yes	Yes	Yes
3	M	85	6.8	Mixed	Mild	TA	–	LP-33S	Yes	No (uncontrolled apical access bleeding leading to death on day 1)	No
4	M	75	3.2	Primary	Severe	TA	–	LP-33S	Yes	Yes	Yes
5	F	88	4.4	Primary	Moderate	TA	–	LP-29L	Yes	Yes	Yes
6	M	84	3.3	Mixed	Mild	TA	–	LP-35 M	Yes	Yes	Yes
7	M	65	2.7	Secondary	None	TA	–	SP-35 M	Yes	Yes	Yes
8	M	87	4	Mixed	Mild	TA	–	SP-35 M	Yes	Yes	Yes
9	M	83	3.6	Primary	Mild	TA	–	LP-35 M	Yes	No (major arrhythmic event leading to death on day 7)	No
10	M	63	6.6	Secondary	Moderate	TA	–	LP-35 M	Yes	No, 4 ( +) MR	Yes
11	F	80	6.8	Mixed	Moderate	TA	–	LP-29S	Yes	Yes	Yes
12	F	59	10.9	Mixed	None	TA	Prior MitralClip	LP-29S	Yes	Yes	Yes
13	M	71	1.7	Primary	None	TA	Physio II (38 mm)	LP-29S	Yes	No (uncontrolled sepsis aggravated right heart failure with major arrhythmic event leading to death on day 13)	No
14	F	83	1	Mixed	None	TA	–	SP-35 M	Yes	Yes	Yes
15	M	80	2	Primary	None	TA	–	SP-37S	Yes	Yes	Yes

Patient #	Sex	Age (years)	Mean MVG	MR-pathology	Access	Procedure type	Prior bioprosthesis/ring	Size (mm)	Implanted prosthesis	Size (mm)	Technical success	Device success	Alive at 30 Days
(b)													
1	F	74	8	Mixed	TA	ViR	Physio II	30	ES XT	26	Yes	Yes	Yes
2	M	45	13	MS	TF	ViV	Perimount	29	ES XT	29	Yes	Yes	Yes
3	F	78	11	MS	TF	ViV	Perimount	27	ES XT	26	Yes	Yes	Yes
4	F	84	10	MS	TF	ViV	Perimount	25	ES XT	23	Yes	Yes	Yes
5	M	79	6	Mixed	TA	ViR	Physio II	28	ES 3	26	Yes	No (major arrhythmic event and ecmo therapy with worsening right heart failure leading to death on day 8)	No
6	M	74	8	Mixed	TA	ViV	Perimount	29	ES 3	29	Yes	Yes	Yes
7	F	46	18	MS	TA	ViV	Perimount	33	ES 3	29	Yes	Yes	Yes
8	M	76	6	Mixed	TF	ViR	Physio II	32	ES 3	29	Yes	Yes	Yes
9	F	70	9	Mixed	TF	ViR	Physio II	30	ES 3	26	Yes	No, 2 ( +) MR	Yes
10	M	56	7	Mixed	TF	ViV	Magna Ease	29	ES 3	29	Yes	Yes	Yes
11	F	72	6	Mixed	TF	ViR	Physio II	28	ES 3	26	Yes	Yes	Yes
12	F	85	28	MS	TF	ViV	Perimount	27	ES 3	26	Yes	Yes	Yes
13	M	83	12	MS	TF	ViV	Perimount	27	ES 3	29	Yes	Yes	Yes
14	F	84	4,3	MR	TF	ViV	Hancock II	29	ES 3	29	Yes	Yes	Yes
15	F	66	15	MS	TF	ViV	Magna Ease	29	ES 3	29	Yes	Yes	Yes
16	M	53	10	MS	TF	ViV	Perimount	31	ES 3	29	Yes	No (therapy refractory ventricular fibrillation leading to death on day 12)	No
17	M	52	6.6	Mixed	TF	ViV	Perimount	31	ES 3	29	Yes	Yes	Yes
18	M	81	3.3	MR	TF	ViR	Physio II	32	ES 3	29	Yes	Yes	Yes
19	M	84	12.2	MS	TF	ViV	Perimount	29	ES 3	29	Yes	Yes	Yes
20	M	78	9.7	Mixed	TF	ViV	CE Standard	31	ES 3	29	Yes	Yes	Yes
21	M	60	10.6	MS	TF	ViV	Magna Ease	27	ES 3	29	Yes	No (MVG: 11 mmHg)	Yes
22	F	84	6.2	Mixed	TF	ViV	Hancock II	27	ES 3	26	Yes	No (stroke)	Yes
23	M	61	9.1	MS	TF	ViMAC	MAC		ES 3	29	Yes	No (reoperation due to severe LVOT obstruction on day 1)	Yes
24	M	73	10	MS	TF	ViV	Perimount	29	ES 3	29	Yes	Yes	Yes
25	F	58	3	MR	TF	ViV	Perimount	33	ES 3	29	Yes	Yes	Yes
26	M	58	3	Mixed	TF	ViR	Physio II	32	ES 3	29	Yes	Yes	Yes
27	M	79	3	MR	TF	ViV	Magna Ease	31	ES 3	29	Yes	Yes	Yes
28	M	81	7.6	Mixed	TF	ViR	Physio I	28	ES 3	26	Yes	No (uncontrolled sepsis leading to multi organ dysfunction syndrome and death on day 18)	No
29	F	90	5.8	Mixed	TF	ViV	CE Standard	27	ES 3 Ultra	26	Yes	Yes	Yes
30	F	81	7.3	Mixed	TF	ViR	Physio II	28	ES 3 Ultra	26	Yes	Yes	Yes
31	F	70	9.7	MS	TF	ViV	Perimount	27	ES 3 Ultra	26	Yes	Yes	Yes

*MAC* mitral annular calcification, MR = mitral regurgitation, *MVG* mitral valve gradient, *TA* transapical, *ES* Edwards Sapien, *LVOT* left ventricular outflow tract, *MR* mitral regurgitation, *MS* mitral stenosis, *MVG* mean mitral valve gradient, *ViR *valve–in–ring, *ViV *valve-in-valve, *ViMAC* valve-in-mitral annular calcification, *TA* transapical, *TF* transfemoral

### Complications and mortality

**Fig. 3:**
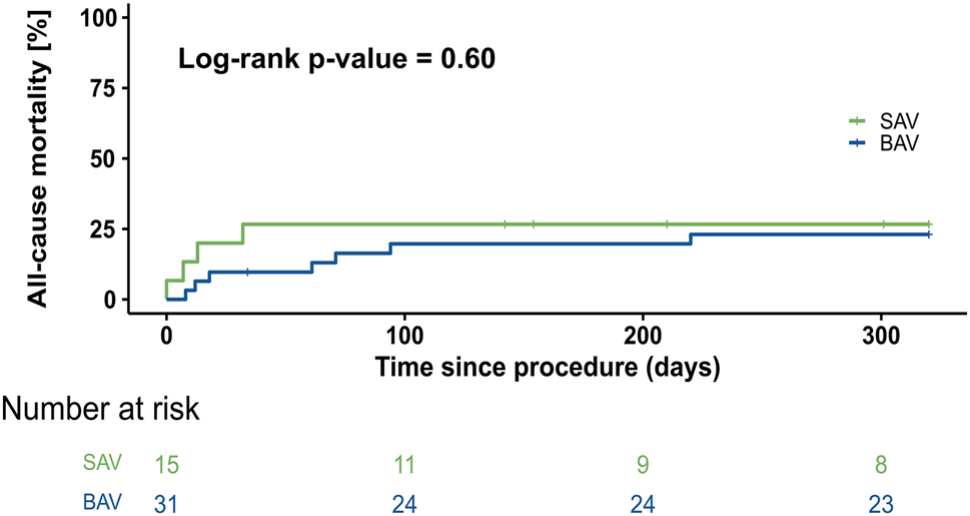
Kaplan–Meier curves showing no difference in rates of all-cause mortality in patients treated either with BAV (balloon-expandable valve) or SAV (self-expanding valve), ticks indicate censored observations. *BAV* balloon-expandable valve, *SAV* Self-expanding valve

No patient needed pacemaker implantation during the follow-up period in both groups. Total hospital stay duration did not differ between both groups. Life-threatening, or major bleeding occurred in 6 patients (40%) in the SAV-group and 4 patients (13%) in the BAV-group (Table 3b). In the SAV-group, one patient suffered from uncontrolled apical access bleeding leading to death on post-operative day 1. Two patients were in critical condition prior to valve treatment and died of cardiovascular causes on post-operative day 7 and 13 (Table 4a). One patient died of COVID pneumonia 32 days after the procedure. After 32 days, no further deaths were observed in the included SAV-group. Overall device success rate at 30 days was achieved in 12 of 15 patients (80%) in the SAV-group and 24 of 31 patients (77.8%) in the BAV-group. One patient with ViMAC had severe LVOT obstruction leading to mitral valve replacement via open heart surgery one day after the interventional procedure. Two patients died of cardiovascular cause and one of non-cardiovascular cause within the first 30 days in the BAV-group (Table 4b). All-cause mortality rates, therefore, were 26.7% and 20% for patients with available follow-up (Fig. 3). No valve migration, embolization or endocarditis were detected.

## Discussion

The current study evaluated the contemporary treatment performance of TMVI either by BAV (SAPIEN-group) or SAV (Tendyne-group) in patients with severe MVD. The key findings can be summarized as follows: (1) In selected high-risk patients, SAV via a transapical approach is a feasible and effective treatment option that offers a valuable option for treatment of MVD, (2) SAV patients show low mean transvalvular gradient after procedure, (3) residual MR was more pronounced among patients undergoing BAV, (4) early after SAV, frail patients in reduced physical condition remain a special challenge with regard to mortality.

A broad spectrum of MVD pathologies can be treated by several options such as medical treatment [17], catheter-based interventions [3] or (redo) surgery [18]. There is a considerable need for treatment options due to increasing use of bioprostheses with high numbers of structural valve degeneration and longer life expectancy. The elevated risk of surgery in this particularly elderly cohort negatively impacts potential beneficial aspects [19]. The use of M-TEER is not applicable in all anatomies, and moderate or severe residual MR is present in around 5–8% of patients following this method [20, 21].

TMVI has emerged as a further alternative for patients with symptomatic MVD. Our current findings support the use of treatment by TMVI with SAV with comparable outcomes to BAV. It is important to note that these treatments are significantly different. Studies describing the direct comparison of BAV and the novel SAV have not been reported, because patients in each group offer different medical and surgical backgrounds.

However, both groups had similarities despite the different rate of prior heart surgery. Compared with the existing literature our cohort includes older patients with a high-risk profile as assessed by the EuroScore II. The risk profile of our present cohort is even higher compared to the largest existing reports for SAV with 6.5 ± 5.0 [22] in the Global Feasibility Study and 8.4 ± 6.1 in the TENDER register [23].

A relevant reduction of MR to less than mild was feasible in the vast majority of our patients undergoing SAV, which is consistent with several reported studies for the Tendyne device [7, 8, 23–25]. One patient experienced moderate–severe MR. This patient had complex anatomy with severe calcification of the mitral annulus.

In the BAV-group, 80% of the patients had none or trace MR, slightly lower compared with previous studies reporting that 90–94% of patients had less than mild MR [5, 6]. Overall experience increases for ViV, ViR and ViMAC with BAV and TMVI with SAV. In addition, consideration of wide anatomical sizes of the mitral annulus, MAC, variability of the sub valvular apparatus and shape of the mitral annulus is crucial for optimal results [26].

In our analysis, we observed a mean transvalvular gradient of 6.4 ± 2.04 in the BAV-group, which was in line with the findings of Simonato et al. [6], and higher compared to Guerrero et al. [15, 27]. The mean gradient in SAV-group was similar to the findings from the TENDER register [23] and slightly higher compared to the selected population in the Global Feasibility Study (2.9 ± 1.3 mmHg) [7].

One possible explanation for this finding could be the number of ViV and ViR cases among the BAV-group finally limiting the comparability of both groups regarding transvalvular gradient.

In the entire cohort LVOT-obstruction was exceptionally low. Using preoperative computerized tomography scan is crucial to prevent this complication. One patient experienced severe LVOT-obstruction after BAV leading to reoperation.

These challenges can be successfully overcome using the intentional anterior mitral leaflet laceration (LAMPOON) technique in patients treated with SAV [28]. In addition, a high transvalvular gradient might translate into a worse outcome as shown for the patient cohort after M-TEER [29]. However, literature concerning this topic is conflicting [30–32].

We added patients with severe TR, which were excluded in the first feasibility study [8] and with only a low number in the TENDER register [23]. PASP in these patients could be reduced highlighting the efficacy of these treatment options. One explanation for the significant reduction was the elevated PASP in the SAV-group. Pulmonary hypertension is frequent among patients with MVD caused by backwards transmission of elevated left atrial pressure. As shown for M-TEER reduction of PASP is feasible and might translate into better survival [33].

Of all patients included into the present analysis, only one patient had a stroke leading to an overall incidence rate of 3.2%. This low incidence rate can be supported with data from other groups [5, 8].

Length of intensive care unit and in-hospital stay were comparable among both groups, whilst overall duration was longer for the BAV compared to the current literature [34].

Given the high-risk profile of this investigated cohort, we observed a relevant post-procedural morbidity and mortality. A total of four deaths occurred early after intervention, 20.0% in the BAV- und 26.7% in the SAV-group. All patients that died in the SAV-group were at very high-risk with no other treatment option. Deaths occurred in the early postoperative phase, a finding that is similarly described in the Global Feasibility Study [7]. Nevertheless all-cause mortality rate was 26% during follow-up, which was in line with the experience of the single-center retrospective study by Ludwig et al. with 33% mortality rate at one year [24]. In contrast, in patients with BAV treatment mortality rate slightly increased after the initial month period. Zubarevich et al. report a 1-year mortality rate of 28%. Furthermore, they found only a minor increase to 37% after three years [35].

The transapical access incorporates a high-risk for the development for life-threatening complications. Of note, transapical access yielded a higher all-cause mortality at one year in comparison with transseptal access (21.7 versus 15.8%) in the TVT registry [36]. However, Nazir et al. did not find a difference regarding the 30-day mortality for both approaches [37]. This is important as the present design of the Tendyne valve does not allow a switch from transapical to transseptal implantation.

But not all complications are access related. Even with variant access strategies in the two distinct patient cohorts in our study, there is a relevant difference between technical success (100% in both groups) and device success at 30 days. Reasons for device failure are mostly patient related and not necessarily device related. Thus, it is important to report and discuss those cases in detail to increase our understanding of prognostic and clinical markers that can help to improve patient outcome and device success in the interventional treatment of complex MVD.

Overall, interventional treatment is superior to medical therapy as evidenced by a low all-cause mortality, which was observed after 1 year in patients either treated with TMVI or M-TEER compared to medical therapy [25].

### Study limitations

This case series has several limitations related to its retrospective design, limited number of included patients and follow-up. The most common exclusion criteria include very severe left ventricular systolic dysfunction, anatomic or size problems and elevated risk of left ventricular outflow tract obstruction. The heterogeneity of our population may have incorporated bias and precludes definitive comparison of TMVI with SAV or BAV, therefore, data are presented as case series. Nevertheless, the present analysis reflects a real-world cohort, which is in our view important to objectively evaluate modern treatment strategies for MVD.

## Conclusion

Under real-world conditions, TMVI with either SAV or BAV yields acceptable midterm outcomes with excellent hemodynamic results with a low incidence rate of paravalvular leak. Therefore, these two therapeutic options complement the existing therapy for MVD.

## Data Availability

The datasets generated during and/or analyzed during the current study are available from the corresponding author on reasonable request.

## Abbreviations

BAV: Balloon-expandable valve
LVOT: Left ventricular outflow tract
MAC: Mitral annular calcification
MR: Mitral regurgitation
MS: Mitral stenosis
MVD: Mitral valve disease
MV: Mitral valve
M-TEER: Mitral transcatheter edge-to-edge repair
PASP: Pulmonary artery systolic pressure
PVL: Paravalvular regurgitation
SAV: Self-expanding valve
TMVI: Transcatheter mitral valve implantation
ViV: Valve-in-valve
ViR: Valve-in-ring
ViMAC: Valve-in-mitral annular calcification
